# Synthesis of Hyperbranched Flame Retardants with Varied Branched Chains’ Rigidity and Performance of Modified Epoxy Resins

**DOI:** 10.3390/polym15020449

**Published:** 2023-01-14

**Authors:** Jingyuan Hu, Liyue Zhang, Mingxuan Chen, Jinyue Dai, Na Teng, Hongchi Zhao, Xinwu Ba, Xiaoqing Liu

**Affiliations:** 1 Ningbo Institute of Materials Technology and Engineering, Chinese Academy of Sciences, Ningbo 315201, China; 2 Key Laboratory of Chemical Biology of Hebei Province, College of Chemistry and Environmental Science, Hebei University, Baoding 071002, China; 3 Key Laboratory of Marine Materials and Related Technologies, Chinese Academy of Sciences, Ningbo 315201, China

**Keywords:** hyperbranched flame retardant, toughness, bio-based, epoxy resin

## Abstract

To overcome the high flammability and brittleness of epoxy resins without sacrificing their glass transition temperature (*T_g_*) and mechanical properties, three epoxy-terminated hyperbranched flame retardants (EHBFRs) with a rigid central core and different branches, named EHBFR-HB, EHBFR-HCM, and EHBFR-HBM, were synthesized. After chemical structure characterization, the synthesized EHBFRs were introduced into the diglycidyl ether of bisphenol A (DGEBA) and cured with 4, 4-diaminodiphenylmethane (DDM). The compatibility, thermal stability, mechanical properties, and flame retardancy of the resultant resins were evaluated. Results showed that all three EHBFRs could significantly improve the fire safety of cured resins, and 30 wt. % of EHBFRs (less than 1.0 wt. % phosphorus content) endowed cured DGEBA with a UL-94 V-0 rating. In addition, the increased rigidity of branches in EHBFRs could increase the flexural strength and modulus of cured resins, and the branches with appropriate rigidity were also beneficial for improving their room temperature impact strength and *T_g_*.

## 1. Introduction

Epoxy resins are widely used as adhesives, coatings, and paints, as well as structural composites, in our daily lives because of their excellent chemical resistance, thermal stability, electrical insulation, and mechanical properties [1]. However, the traditional epoxy, i.e., diglycidyl ether of bisphenol A (DGEBA), is highly flammable and brittle, which greatly restricts its application fields [2,3].

A variety of flame retardants, including phosphorus, nitrogen, silicon, and metal-containing compounds, have been applied to improve the fire safety of epoxy resins [2,4]. Traditionally, the incorporation of halogen-containing compounds was considered an appealing method to endow epoxy resins with improved flame resistance, due to the low cost, high flame-retardant efficiency, and little negative effects on the thermal or mechanical properties of target products [5]. However, concerns about the persistence and accumulation of halogenated flame retardants, combined with the release of toxic and corrosive fumes during combustion, have already resulted in strict restrictions on their application fields [6]. Currently, phosphorus-containing compounds have been regarded as one of the most promising flame retardants because of their chemical versatility, safety, and high flame retardancy. Among them, 9, 10-dihydro-9-oxa-10-phosphaphenanthrene-10-oxide (DOPO) and its derivatives have been widely used [7]. Wang et al. [8] once prepared a new kind of reactive organophosphorus flame retardant, DPDDM, from DOPO. Compared with the pristine epoxy, the modified epoxy resin presented better mechanical properties and flame retardancy. Zhang et al. [9] reported that the flame retardancy of epoxy could be greatly improved by the introduction of DPE (a high-efficiency DOPO-based reactive flame retardant synthesized by the group). Unfortunately, the introduction of rigid DOPO units led to decreased cross-linking density, thereby damaging the *T_g_* of cured resins. Moreover, the phenanthrene ring of DOPO will further increase the brittleness and crack sensitivity of epoxy resin due to the poor mobility of the DOPO-containing segment, which makes it difficult to conduct the energy transfer when subjected to impact, and then stress concentration occurs [10,11].

In addition, the epoxy resins also present high brittleness, and their toughness improvement is of great significance and highly desired [12,13]. Hyperbranched polymers (HBP) have been proven to be very effective toughening agents [14,15] since they contain a large number of intramolecular holes, which can provide more opportunities and space for the movement of molecular segments under stress and then improve the toughness of polymeric materials [16,17,18]. Moreover, to simultaneously improve the flame retardancy and toughness of epoxy resins, the flame retardants have been introduced into HBPs, named hyperbranched flame retardant (HBFR), to give epoxy resins simultaneously improved toughness and flammability [19,20,21,22,23]. However, the synthesized HBFR usually leads to a decreased *T_g_* because of the great number of intramolecular cavities and flexible chain. To maintain the *T_g_* of epoxy resin, rigid aromatic rings were incorporated into HBFR, and modified epoxy resin showed elevated *T_g_* [24]. Unfortunately, their modulus and strength were usually depressed [25]. How to simultaneously overcome the flammability and brittleness of epoxy resins without sacrificing their *T_g_* and mechanical properties has always been a significant issue. In addition, to the best of our knowledge, little attention has been paid to the synergistic effect of HBFR’s chemical structure and topological structure in hyperbranched systems. How HBFR affects the *T_g_*, strength, and modulus of cured epoxy resin while improving the toughness and flame retardancy of epoxy resin systems has rarely been reported.

**Scheme 1:**
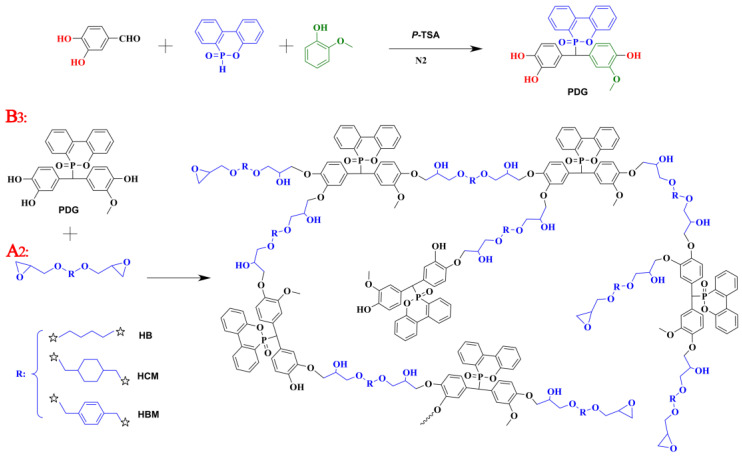
Synthetic routes of EHBFR-HB, EHBFR-HCM, and EHBFR-HBM.

In this work, the bio-based protocatechualdehyde and guaiacol, together with DOPO, were used to prepare the epoxy-terminated hyperbranched flame retardants (EHBFRs) for DGEBA modification. In order to investigate the influence of EHBFRs’ branch rigidity on the properties of modified DGEBA resin in terms of flame-retardancy, toughness, strength, and *T_g_*, three EHBFRs with a rigid central core (DOPO) and branches with different rigidities (EHBFR-HB, EHBFR-HCM, and EHBFR-HBM) were designed and synthesized (Scheme 1). By characterizing the crosslink density and free volume fraction of the modified epoxy resin, the effect of the rigidity of the hyperbranched structure on the thermal and mechanical properties of the cured modified epoxy compound was elucidated.

## 2. Materials and Methods

### 2.1. Raw Materials

Protocatechuic aldehyde (PCAD, 98.0%), p-toluenesulfonic acid (p-TSA, 98.5%), guaiacol (98.0%), epichlorohydrin (ECH, 99.0%), 1,4-butanediol (98.0%), 1,4-cyclohexanedimethanol (99.0%), p-phenylenedimethanol (99.0%), tetrabutyl ammonium bromide (TBAB, 99.0%), dimethyl sulfoxide (DMSO, 99.9%) and 4, 4-diaminodiphenylmethane (DDM, 98.0%) were obtained from Aladdin Reagent, shanghai, China. 9, 10-dihydro-9-oxa-10-phosphaphenanthrene-10-oxide (DOPO, 98.0%) were supplied by Guizhou Yuanyi Mining Group Co., Ltd.Guiyang, China. Sodium hydroxide (96.0%), chloroform (99.0%), ethanol (95.0%), and petroleum ether (99.8%) were acquired from Sinopharm Chemical Reagent Co., Ltd., shanghai, China. DGEBA (DER331, an epoxy with a value of 0.51–0.53 mol/100 g) was obtained from Dow Chemical Company, shanghai, China. All the solvents and reagents were used as received, without further purification.

PDG was synthesized from DOPO, protocatechuic aldehyde, and guaiacol following the protocol reported in previous literature [26]. The chemical structure of PDG was confirmed by FT-IR and NMR, as presented in Supplementary Materials Figure S1.

### 2.2. Synthesis of Diglycidyl Ether

1, 4-butanediol (7.21 g, 0.08 mol), tetrabutyl ammonium bromide (0.66 g), and 100 mL of a 40 wt.% sodium hydroxide solution were added to a round bottom flask with a magnetic stirrer. The mixture was maintained at 50 °C for 1 h. Then the reaction mixture was cooled with an ice bath, and slowly 74.02 g (0.8 mol) of ECH was added. Subsequently, it was heated to 25 °C and kept for 24 h. After that, 150 mL of chloroform was added to the reaction solution, and the organic phase was washed alternately with 1 mol/L sodium hydroxide solution and deionized water for several times and dried with anhydrous sodium sulfate. Then the extraction was evaporated under reduced pressure to remove the solvent and ECH. Finally, the colorless liquid was obtained with a yield of 83.1%.

1, 4-cyclohexanedimethanol diglycidyl ether and p-phenylenedimethanol diglycidyl ether were prepared by a synthetic step similar to 1, 4-butanediol diglycidyl ether. The obtained products were colorless liquids with a yield of 81.5% and 84.7%, respectively.

### 2.3. Synthesis of EHBFR-HB, EHBFR-HCM, and EHBFR-HBM

Three kinds of epoxy terminated hyperbranched flame retardants (EHBFRs) were prepared by A_2_ + B_3_ polymerization with 1,4-butanediol diglycidyl ether, 1,4-cyclohexanedimethyldiglycidyl ether, and p-phenyldimethyldiglycidyl ether as A2 monomers and PDG as B3 monomers, named EHBFR-HB, EHBFR-HCM, and EHBFR-HBM, respectively. The synthesis route was shown in Scheme 1. EHBFR-HB was synthesized using 1, 4-butanediol diglycidyl ether (40.45 g, 0.20 mol) as the raw material and TBAB (1.90 g, 3 wt.% of the mixture) as the catalyst in a round bottom flask equipped with a magnetic stirrer and a reflux condenser. After the mixture was uniform and transparent with 50 mL DMSO, PDG (23.01 g, 0.05 mol) dissolved in 50 mL DMSO was added drop by drop to the round bottom flask and the mixture was maintained at 100 °C for 12 h. Subsequently, the mixture was cooled to room temperature and extracted by 200 mL of chloroform. The extraction was washed alternately with a sodium hydroxide solution and deionized water for several times and dried by anhydrous magnesium sulfate. Then, it was poured into petroleum ether and dried under reduced pressure. Finally, reddish brown viscous liquid (EHBFR-HB) was obtained with a yield of 80.6%.

EHBFR-HCM was synthesized in similar steps using 1, 4-cyclohexane dimethyl diglycidyl ether and PDG. EHBFR-HBM was synthesized in similar steps using p-phenyldimethyl diglycidyl ether and PDG. Finally, light yellow fine powder (EHBFR-HCM and EHBFR-HBM) was obtained with yields of 81.7% and 84.3%, respectively.

### 2.4. Preparation of Flame-Retardant Epoxy Thermosets

Epoxy thermosets were prepared from epoxy-terminated hyperbranched flame retardants (EHBFRs, including EHBFR-HB, EHBFR-HCM, or EHBFR-HBM), DGEBA, and stoichiometric curing agent (DDM), and the detailed compositions are presented in Table 1. The mixtures of DGEBA and various contents of EHBFR-HB, EHBFR-HCM, or EHBFR-HBM were stirred at 120 °C until they were transparent and homogeneous, and then a certain amount of DDM was added to the liquid mixtures. After being degassed under vacuum for 5 min, the mixtures were poured into molds and then step-cured in an oven with the following heating processes: 120 °C for 2 h, 140 °C for 2 h, 160 °C for 2 h, and 180 °C for 2 h. After that, all the samples were slowly cooled down to room temperature and carefully removed from the molds.

### 2.5. Measurements

Fourier transform infrared (FT-IR) spectra were recorded by a NICOLET 6700 spectrometer (Thermo-Fisher, US) with KBr pellets in the range of 400 to 4000 cm^−1^. ^1^H, ^13^C, and ^31^P nuclear magnetic resonance (NMR) spectra were acquired using a 400 MHz AVANCE III Bruker NMR spectrometer (Bruker, Zurich, Switzerland) in either DMSO-d6 or CDCl_3_ with tetramethysilane (TMS) as an internal standard. Gel permeation chromatography (GPC; Waters 1515, Waters, Milford, MA, USA) was used to determine the molecular weight and molecular weight distribution of EHBFR-HB, EHBFR-HCM, or EHBFR-HBM with DMF as the eluent; calibration was based on a series of narrow molecular weight linear polystyrene. The mass spectrometry was conducted by LC-Q-TOF (AB Sciex, Boston, MASS, USA). The epoxy value of EHBFR-HB, EHBFR-HCM, or EHBFR-HBM was determined by a hydrochloric acid-acetone titration method under the standard GB1677-81. The content of phosphorus in hyperbranched flame retardants was determined by inductively coupled plasma emission spectrometry. In order to determine the curing process and analyze the curing behavior, differential scanning calorimetric (DSC) measurements were conducted on a Mettler-Toledo MET DSC (METTLER TOLEDO, Zurich, Switzerland) at heating rates of 5, 10, 15, and 20 °C/min from 25 to 250 °C. DSC measurements were also carried out to determine the *T_g_* of cured resins, and the values were obtained from the second heating rate curve at heating rates of 20 °C/min. All the DSC analyses were performed under a high-purity nitrogen atmosphere with a flow rate of 20 mL/min. Thermogravimetric analyses (TGA) were carried out using a Mettler-Toledo TGA/DSC1 (METTLER TOLEDO, Zurich, Switzerland) under a nitrogen atmosphere at a heating rate of 20 °C/min from 50 to 800 °C. Raman mapping was obtained with a confocal micro-Raman spectrometer (Renishaw in Via Reflex, Renishaw, London, UK) using a 785 nm laser. The mechanical properties of epoxy composites were determined with a universal tester (Instron 5567, Instron, Shanghai, China) and a GT-7045-HML impact tester (GOTECH, Guangzhou, China) with a 1.0 J pendulum. The three-point flexural tests were performed at 25 °C with a rate of 2 mm/min under the standard ASTM D790. The notched impact property was separately characterized at 25 °C and −196 °C, respectively, under the standard ASTM D-256. Dynamic mechanical analyses (DMA) were carried out with a DMA Q800 instrument (TA, New Castle, DE, USA) in tension mode with a 3 °C/min heating rate from −20 to 250 °C at a frequency of 1 Hz, and the sample size is 20 × 5 × 0.5 mm^3^. Thermomechanical analyses (TMA) were carried out on a TMA402 F3 tester (NETZSCH, Bavaria, Germany) with a heating rate of 3 °C/min. The UL-94 tests were carried out according to ASTM D3801 on a CZF-3 instrument (Jiangning Analysis Instrument Company, Jiangning, China) with a sample dimension of 130 × 13.0 × 3.0 mm^3^. The LOI values were obtained using an HC-2 Oxygen Index instrument (Jiangning Analytical Instrument Co., Ltd., Jiangning, China) with a sample size of 130 × 6.5 × 3.2 mm^3^ following ASTM D2863-97. MCC-2 (Suzhou Yangyi Vouch Testing Technology Co. Ltd., Suzhou, China) was used to investigate the combustion of epoxy thermosets, and about 5 mg sample was heated from 70 to 700 °C at a heating rate of 1 °C/s under nitrogen following ASTM D2863-97. The MCC test can obtain the heat release rate (HRR) from the amount of oxygen loss, and the total heat release (THR) can be calculated by integrating the heat release rate over time. It can be used to evaluate flame retardancy, which requires fewer samples and gives more accurate results. The morphology of fractured surfaces after notched impact tests and LOI tests was investigated bya S4800 scanning electron microscope (SEM; Hitachi, Tokyo, Japan), and all samples were sputtered with about a 10 nm gold layer before being tested. 

## 3. Results and Discussion

### 3.1. Synthesis and Characterization of EHBFR-HB, EHBFR-HCM, or EHBFR-HBM

**Figure 1:**
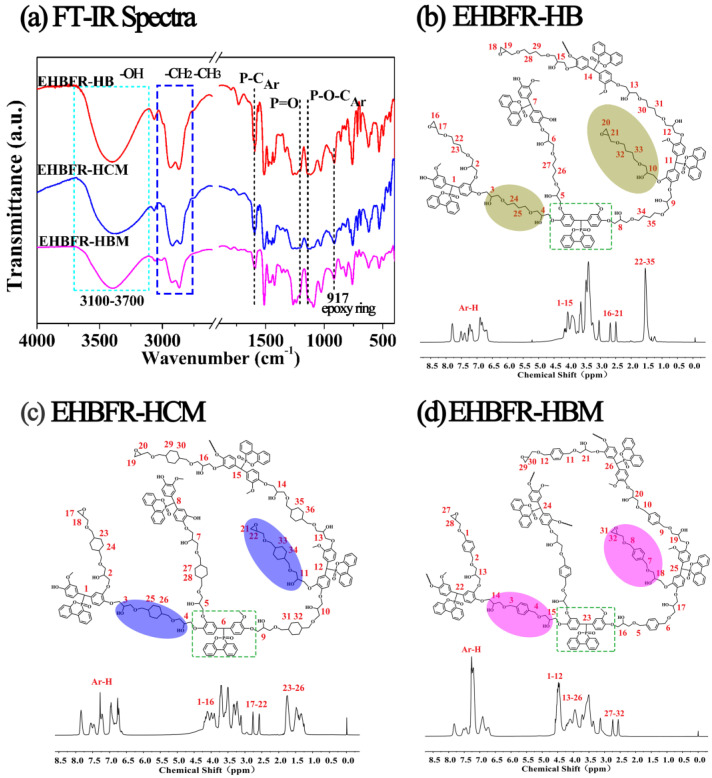
FT-IR spectra of EHBFR-HB, EHBFR-HCM, and EHBFR-HBM (**a**) and ^1^H NMR spectra of EHBFR-HB (**b**), EHBFR-HCM (**c**), and EHBFR-HBM (**d**).

In this study, PDG was prepared from DOPO, protocatechuic aldehyde, and guaiacol in one step. The chemical structure of PDG was confirmed by FT-IR and NMR, as presented in Figure S1. Epoxy terminated hyperbranched flame retardants (EHBFRs) were obtained by typical A_2_ + B_3_ polymerization, and FT-IR characterization results were shown in Figure 1a. Compared with PDG, the wide absorption band appearing at 3100–3700 cm^−1^ in Figure 1a replaced the sharp absorption peak showing at 3505 cm^−1^ in Figure S1, illustrating the formation of the alcohol hydroxyl group due to the chemical reaction between the phenolic hydroxyl group in PDG and the epoxy group. Besides, for the three EHBFRs in Figure 1a, there appeared the characteristic absorption peaks of phosphophenanthrene groups and that of near 917 cm^−1^ for epoxy groups [26]. Meanwhile, according to Figure 1(b-d), the chemical shifts of protons were in good agreement with the chemical structures of EHBFR-HB, EHBFR-HCM, and EHBFR-HBM. All these results indicated the successful synthesis of EHBFR-HB, EHBFR-HCM, and EHBFR-HBM. The epoxide values (EV/mol/100 g) of EHBFR-HB, EHBFR-HCM, and EHBFR-HBM determined by HCl-acetone standard titration were 0.15, 0.13, and 0.14, respectively, which suggested the three EHBFRs had a similar EV of about 0.14.

The molecular weights (Mn), polydispersity index (PDI), as well as their branching degree (DB) of hyper-branched polymers have an important effect on the properties of the modified materials. As can be seen in Table 2, the three EHBFRs had similar Mn (about 4000 g/mol), PDI (about 2), and DB (about 0.7). In all, the above results revealed that the characteristics of EHBFR-HB, EHBFR-HCM, and EHBFR-HBM were similar, except for their branch chain rigidity.

### 3.2. Processability and Compatibility

Traditionally, the introduction of EHBFRs would greatly inhibit the reaction of DGEBA epoxy resin with amine curing agent [27] due to the limited diffusion of reactive groups due to the steric hindrance of hyperbranched polymers, while the introduction of hydroxyl could improve the curing activity of DGEBA epoxy resin with amine curing agent [28]. In this work, EHBFR-HB, EHBFR-HCM, and EHBFR-HBM are hyperbranched polymers containing hydroxyl groups, so it is necessary to explore their effects on the reactivity between DGEBA and DDM. To assess the curing reactivity, the overall polymerization reaction kinetics of different mixture systems were investigated by DSC under non-isothermal conditions at various heating rates of 5, 10, 15, and 20 °C/min. As shown in Figures S2–S4, compared with pure DGEBA, the samples with different contents of EHBFRs displayed a slightly lower curing peak temperature, revealing that the introduction of EHBFRs (EHBFR-HB, EHBFR-HCM, and EHBFR-HBM) enhanced the curing reactivity of DGEBA and DDM. Furthermore, the apparent activation energies (*E_a_*) of all the mixtures were calculated based on the Kissinger (1) and Ozawa (2) methods [29,30]:

(1) $$-\ln(\beta /T_{p}{}^{2})=E_{a}/RT_{p}-\ln(AR/E_{{}^{a}})$$

(2) $$\ln\beta =-1.052*E_{a}/RT_{p}+\ln(AE_{a}/R)-\ln(F(x))-5.331$$

where β is the heating rate, *T_p_* is the absolute exothermic peak temperature, and *R* is the ideal gas constant. From the slope of the plots of ln (β/*T_p_^2^*) and ln β versus 1/*T_p_*, *E_a_* is the curing activation energy for the curing systems that were determined. 

**Figure 2:**
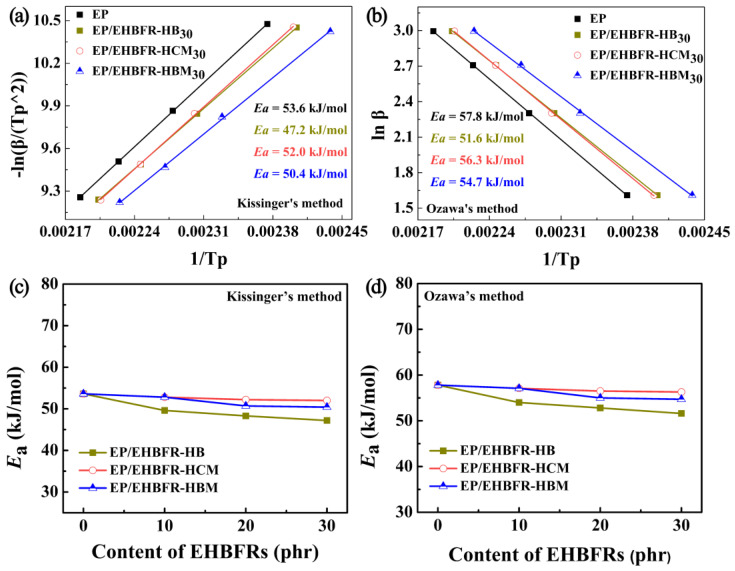
Plots generated following the Kissinger’s (**a**,**c**) and Ozawa’s (**b**,**d**) methods for determination of the activation energy of epoxy thermosets.

As displayed in Figure 2, the DGEBA system shows *E_a_* values of 53.6 and 57.8 kJ/mol, respectively, which agrees well with previous studies [26,31]. All the EHBFRs-modified epoxy resin samples gave a lower curing activation energy relative to that of pristine DGEBA, indicating some promotion for EHBFRs in the curing reaction of epoxy resin. Meanwhile, the EP/EHBFR-HB hybrid system showed the highest curing activity among the three EP/EHBFRs hybrid systems due to its lowest curing activation energy. It may be the less rigid molecular chain of EHBFR-HB that is conducive to the movement of chain forging, which increases the collision probability between the epoxy group and curing agent and thus improves the curing reaction rate [32].

The compatibility between components of cured resins has an important impact on their thermal and mechanical properties. The microstructure of epoxy cured products modified by different EHBFRs was characterized by the Raman test. Figure S5 gives the Raman spectra of EHBFR-HB, EHBFR-HCM, EHBFR-HBM, and pure DGEBA. Compared with DGEBA, it was observed that the individual characteristic peaks of EHBFRs were at 1050 cm^−1^, belonging to the stretching vibration of P-O-C [33]. Therefore, the Raman images were generated by plotting the integral area of the scattering band at 1050 cm^−1^ as a function of position, to illustrate the distribution of EHBFRs in DGEBA for EHBFRs/DGEBA (EP/EHBFR-HB30, EP/EHBFR-HCM30, and EP/EHBFR-HBM30) blends and the corresponding cured system. It is well known that the laser light source will not move with the fluctuation of the sample surface [26]. Therefore, the laser light source may not focus on the surface with the change in surface conditions when the sample surface roughness is large, resulting in signal weakening and a baseline increase. In order to obtain more accurate data, a microscope was employed to select a flat sample surface.

**Figure 3:**
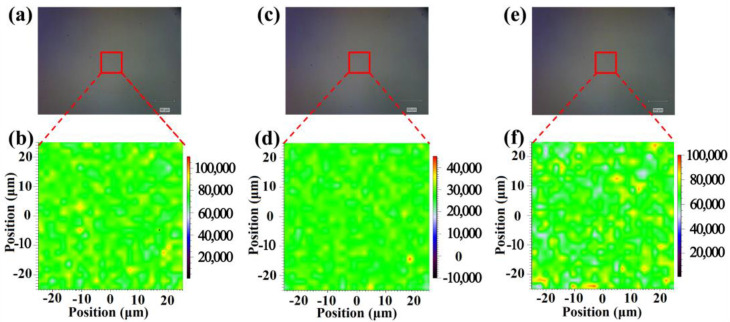
The video and Raman images of cured EP/EHBFR-HB30 (**a**,**b**), cured EP/EHBFR-HCM30 (**c**,**d**), and cured EP/EHBFR-HBM30 (**e**,**f**).

As shown in Figure 3a,c,e, the cured samples of EP/EHBFR-HB30, EP/EHBFR-HCM30, and EP/EHBFR-HBM30 were flat and could be used as the mapping scanning area. Figure 3b,d,f were Raman images of the selected areas in Figure 3a,c,e), respectively. The brighter portion indicates the higher content of EHBFRs, while the darker portion suggests the higher content of DGEBA. Obviously, the color distribution in Figure 3b,d,f was uniform, with few bright areas and dark areas, indicating that EHBFRs were evenly distributed in DGEBA, that was, they showed good compatibility with the DGEBA epoxy. Moreover, EP/EHBFR-HB30, EP/EHBFR-HCM30, and EP/EHBFR-HBM30 cured resins displayed homogeneous structures in their Raman images.

### 3.3. Thermal Stability of Cured Resins

**Figure 4:**
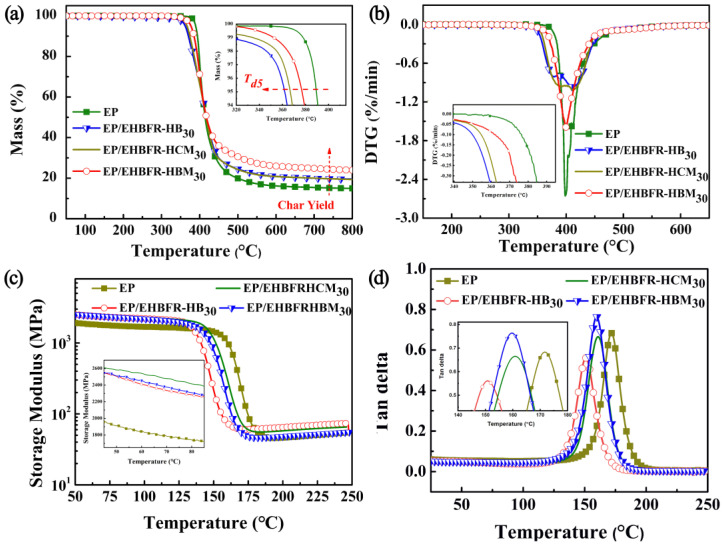
Non-isothermal TGA and DTG curves (**a**,**b**) for cured EP/EHBFR30; variation of storage modulus with temperature for cured EP/EHBFR_30_ (**c**); and *T_g_* of cured EP/EHBFR_30_ (**d**).

Due to the poor thermal stability of the O=P-O bond, the DOPO-containing polymers generally decompose at relatively low temperatures [34]. Figure S6 displays the mass loss of epoxy composites (TGA and DTG) as a function of temperature under a nitrogen atmosphere. As observed, the introduction of EHBFRs did not change the degradation process of the epoxy resin, and all the cured epoxy resins exhibited the same degradation behavior as the pure EP did. This process consists of three stages: from 50 to 350 °C, the mass shows little change as the temperature rises, and the second stage (from 350 to 550 °C) shows a gradual decrease in mass as the temperature continues to rise. In the third stage, as the temperature rises from 550 to 800 °C, there is almost no loss of mass. Compared with pristine EP, all the cured systems containing EHBFRs performed at lower initial decomposition temperatures, and T_d5_ decreased slightly with the increase of the content of EHBFRs, which was in congruence with previous studies that indicated the decomposition of DOPO-containing moieties promoted the initial degradation of epoxy resins [35]. As shown in Figure 4a, relative to those of EP/EHBFR-HB_30_ and EP/EHBFR-HCM_30_, the cured product of EP/EHBFR-HBM_30_ showed a higher thermal stability, which may be related to the presence of more benzene rings in the EHBFR-HBM chemical structure. The charring ability of polymers is crucial to their flame-retardant properties. It can be seen from the DTG curve (Figure 4b) that the introduction of EHBFRs significantly reduced the maximum decomposition rate (R_max_) of epoxy-cured products. The reduction of R_max_ is conducive to the formation of a stable carbon layer, which may improve the flame-retardant performance of epoxy cured products. All these results indicated that the fire safety of EHBFR-containing systems had improved. The thermal stability of cured products was gradually increased as the rigidity of EHBFRs increased.

### 3.4. Thermal and Mechanical Properties

Figure S7 and Figure 4c,d showed the DMA curves of pristine EP and modified systems. As can be seen in Figure S7, *T_g_* of the three EP/EHBFRs systems showed different trends with the increase in EHBFRs. *T_g_* of the EP/EHBFR-HB systems decreased with an increase in EHBFR-HB content. However, with the increase of EHBFR-HCM and EHBFR-HBM content, the *T_g_* of EP/EHBFR-HCM and EP/EHBFR-HBM systems first increased and then decreased. Generally, increasing the rigidity of polymer chain forging should improve its *T_g_*. However, EP/EHBFR-HCM_30_ system cured product gave a higher *T_g_* compared with EP/EHBFR-HB_30_ and EP/EHBFR-HBM_30_, although the rigidity of the epoxy resin was not maximum. It may be because the cross-linking density of cured products, as well as the free volume fraction of cured product chain forging, also greatly affects their *T_g_*. According to the theory of rubber elasticity, the cross-linking density of cured resins was calculated by the following equation [36], and related values were summarized in Table 3:

(3) $$V_{e}\prime =E\prime /3RT$$

where *E′* is the storage modulus of epoxy thermosets in the rubbery plateau region, *R* is the universal gas constant, and *T* is the absolute temperature. In this work, the introduction of EHBFRs increases the crosslinking density of epoxy-cured products. Under the same EHBFR content, the crosslinking density of EP/EHBFR-HB systems was the highest, while that of EP/EHBFR-HBM systems was the lowest. The crosslinking density of EP/EHBFR-HB systems increased with increasing EHBFR-HB content. The crosslinking density of EP/EHBFR-HCM and EP/EHBFR-HBM systems increased first and then decreased as the content of EHBFRs (EHBFR-HCM or EHBFR-HBM) rose, reaching the maximum when the content of EHBFRs was 20 wt.%. According to the Flory theory [37], the free volume can be calculated based on the following formula:

(4) $$f_{T}=f_{g}+\Delta \alpha (T-T_{g})$$

(5) $$\Delta \alpha =\alpha_{r}-\alpha_{g}$$

where, *f_T_* is the free volume at temperature *T* °C, *f_g_* is the free volume at *T_g_*, *α_r_* is the coefficient of the expansion at the rubber state, and *α_g_* is the coefficient of the expansion at the glass state. Generally, the cured epoxy resins are regarded as isotropic materials, so the value of the coefficient of expansion is triple that of the linear coefficient of thermal expansion (LCTEs). The LCTEs of epoxy thermosets modified with EHBFRs were measured by TMA, and the results are listed in Table 3.

It can be seen that Δ*α* of EP/EHBFRs all increased with the increasing content of EHBFRs, indicating that the introduction of EHBFRs made the cured epoxy have a larger free volume. In addition, under the same content, the free volume fractions of EP/EHBFR-HBM systems were slightly higher than those of EP/EHBFR-HB and EP/EHBFR-HCM systems, and those of EP/EHBFR-HB systems were the least. Therefore, the *T_g_* of the EP/EHBFR-HCM and EP/EHBFR-HBM systems decreased with the increase of the EHBFRs’ addition content when the addition amount of EHBFRs exceeded 10 wt.%, which was mainly related to the increase of the free volume fraction of chain forging of the cured epoxy resin arising from the decrease of its crosslinking density. In addition, the *T_g_* of EP/EHBFRs did not increase as EHBFRs’ structural rigidity rose, which was mainly related to the incomplete curing caused by too much rigidity and too much free volume fraction.

**Figure 5:**
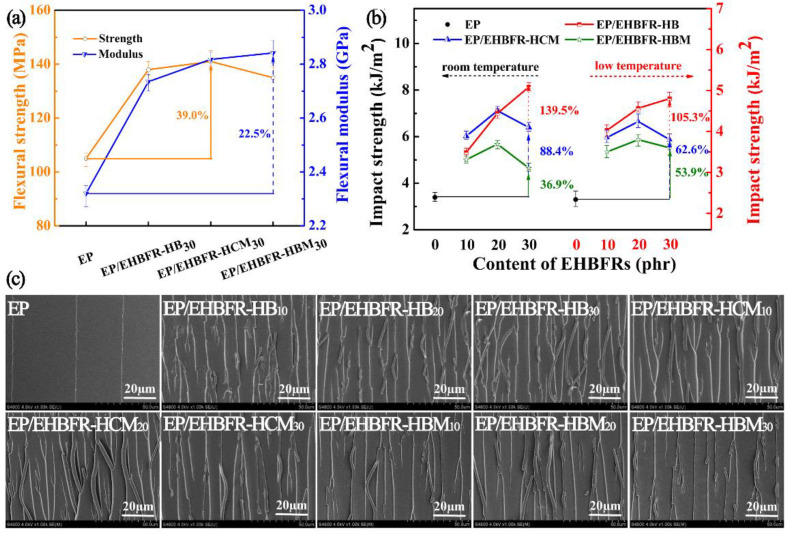
Flexural properties (**a**), impact strength (**b**) and SEM images of fracture surfaces (**c**) for epoxy thermosets after impact strength tests.

The effects of EHBFRs with different rigidities on the mechanical properties of cured products were further studied by flexural and impact tests. The flexural properties of pure EP and EP/EHBFRs systems are shown in Figure S8. As shown, with the increase of EHBFRs, the flexural strengths of EP/EHBFR-HB and EP/EHBFR-HCM systems increased, while the flexural strengths of EP/EHBFR-HBM systems increased with the increase of EHBFR content. By contrast, that of EP/EHBFR-HBM systems displayed a tendency to increase and then decline. As is known to all, the flexural strength is mainly related to the structural rigidity and crosslink density. In addition, the increase of flexural modulus was accompanied by an increase in EHBFR content, which was similar with the change trend of the storage modulus at 25 °C. As shown in Figure 5a, the flexural modulus of EP/EHBFR-HBM_30_ system was the highest, while that of the EP/EHBFR-HB_30_ system was the lowest, which may be attributed to the highest rigidity of EP/EHBFR-HBM_30_.

Generally, the introduction of EHBFRs can greatly improve the toughness of epoxy resin, on the contrary, the introduction of DOPO units increases their brittleness [10,14]. In this work, the DOPO unit was introduced into the branched chain of hyperbranched polymers to explore its impact on the toughness of cured epoxy resin. The toughness was measured by room temperature and low-temperature notch impact strength. As shown in Figure 5b, the room temperature and low temperature impact strengths of EP/EHBFR-HCM and EP/EHBFR-HBM systems increased significantly at first and then decreased with the increasing content of EHBFRs. However, in the case of EP/EHBFR-HB systems, its room temperature and low temperature impact strength increased greatly. The toughness of epoxy was closely related to the free volume fraction; therefore, the introduction of EHBFR-HB, EHBFR-HCM, and EHBFR-HBM greatly improved the impact strength of epoxy. As well, the molecular chain rigidity and crosslink density of epoxy resins have a great impact on their impact strength. Generally, polymers with large flexibility display better impact resistance, while rigid chain materials show the opposite results. Meanwhile, high crosslinking density leads to the brittleness of the material [38]. Due to the synergistic effects of free volume fraction, molecular chain rigidity, and crosslink density, EP/EHBFR-HB_30_ showed the highest room and low temperature impact strengths, which were 2.39 and 2.05 times higher than those of pure EP systems, respectively.

The SEM morphology of the cross-section after the impact test of the pristine EP system and EP/EHBFR systems is shown in Figure 5c. Obviously, pristine EP gave a smooth cross-section, indicating its typical brittle fracture; by contrast, EP/EHBFR systems exhibited a rough cross-section with a large number of filaments. The filaments are caused by the amount of energy that the sample absorbed during the impact, resulting in higher toughness. Therefore, the presence of these filaments further confirmed the excellent toughness of modified epoxy-cured products.

### 3.5. Flame-Retardant Performance

**Figure 6:**
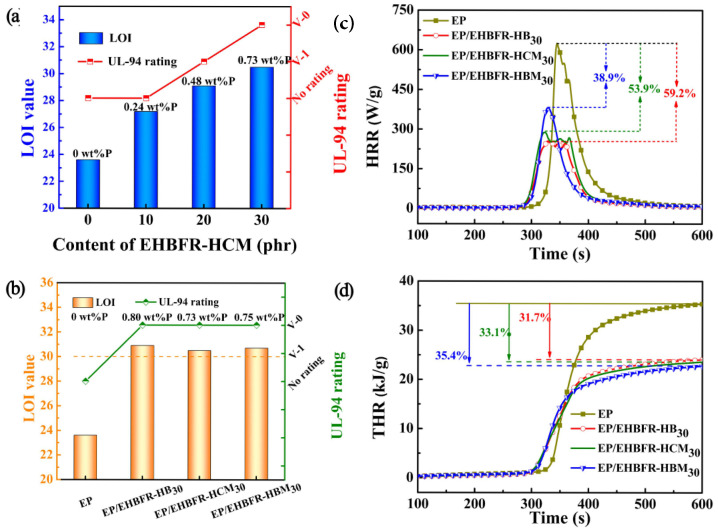
The LOI and UL-94 rating of EP/EHBFR-HCM (**a**), the LOI and UL-94 rating of EP/EHBFR30 (**b**), the HRR (**c**), and the THR (**d**) curves of epoxy thermosets.

**Figure 7:**
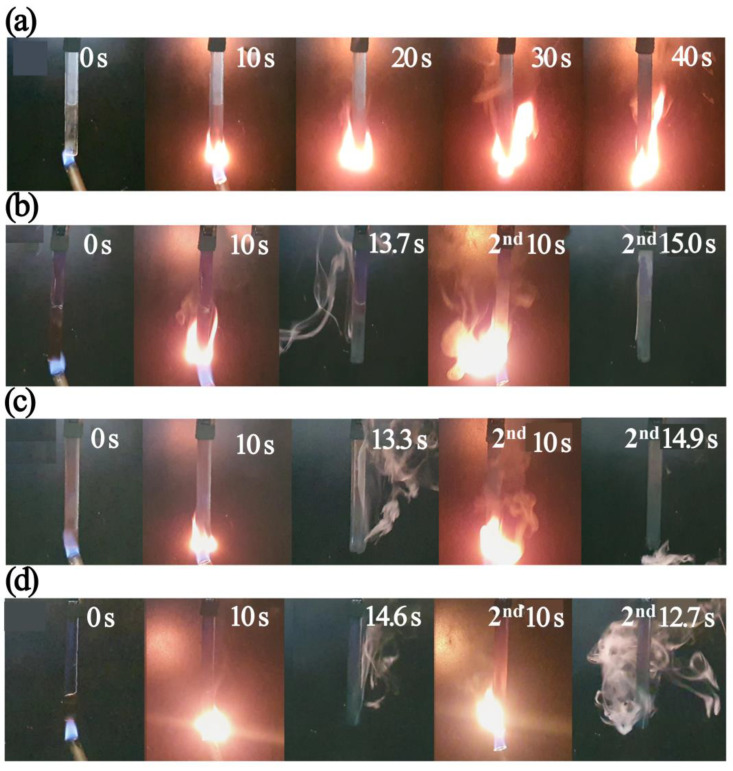
Digital photographs of EP (**a**), EP/EHBFR-HB_30_ (**b**), EP/EHBFR-HCM_30_ (**c**), and EP/EHBFR-HBM_30_ (**d**) during the vertical burning test.

LOI, UL-94 vertical-burning, and MCC tests were used to investigate the effect of EHBFRs on inhibiting the flammability of EP, and detailed results were collected in Table S1 and Figure 6. Apparently, the cured, pristine EP was inflammable, with LOI values of 23.6% and serious flaming drips and smoke in the combustion process (Figure 7). With the increasing content of EHBFRs, the LOI of EP/EHBFR systems increased significantly; take the EP/EHBFR-HCM as an example in Figure 6a. Besides, comparing the same content of EHBFRs, the LOI increased to 30.9% for EP/EHBFR-HB30, 30.5% for EP/EHBFR-HCM30, and 30.7% for EP/EHBFR-HBM30 from 23.6% for EP, as shown in Figure 6b. With regard to the flame retardant grade, UL-94 V-0 ratings were scored for EP/EHBFR-HB30, EP/EHBFR-HCM30, and EP/EHBFR-HBM30 (Figure 7).

MCC has the capability to measure the heat release rate (HRR) based on the oxygen consumption history, and the total heat release (THR) can be calculated from the time integral of the heat release rate (Figure S9). As displayed in Figure 6c,d, the peak heat release rate (pk-HRR) of cured resins dramatically reduced from 625 W/g for EP to 255 W/g for EP/EHBFR-HB_30_, 288 W/g for EP/EHBFR-HCM_30_, and 382 W/g for EP/EHBFR-HBM_30_. Moreover, the THR values also follow the descending order from EP to 24.1 KJ/g for EP/EHBFR-HB_30_, 23.6 KJ/g for EP/EHBFR-HCM_30_ and 22.8 KJ/g for EP/EHBFR-HBM_30_. All these MCC results demonstrated that EHBFRs successfully reduced the fire hazard of epoxy resin.

**Figure 8:**
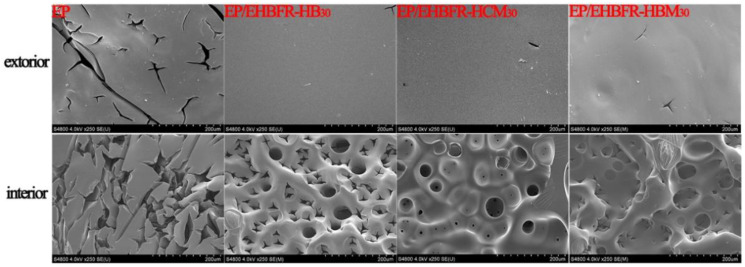
SEM micrographs of char residues after the UL-94 test: external char residues and internal char residues.

The SEM micrographs of both external and internal char residues after the UL-94 test are shown in Figure 8. There were many cracks on the external char layer of pure EP. Contrarily, the EP/EHBFR systems (EP/EHBFR-HB_30_, EP/EHBFR-HCM_30_, and EP/EHBFR-HBM_30_) show compact and continuous surfaces. The strong and sealed surface is expected to effectively diminish the release of flammable volatiles and the transfer of heat. In addition, as shown in Figure 8, EHBFRs obviously contributed to forming internal char residues with honeycomb-like structure, which is conducive to reduce the heat transfer. Therefore, it’s deduced that the sealed exterior char layers, together with honeycomb-like interior char residues, contributed to the flame safety of EP/EHBFR systems.

In a word, the introduction of EHBFR endowed the cured product with better flame retardancy, toughness, higher strength, and *T_g_*. In order to highlight the effect of EHBFRs, a comparison of the increment in toughness, strength (the superscript a is flexural strength, and the superscript b is tensile strength), and *T_g_* of epoxy that can reach UL-94 V-0 reported in the literature is shown in Table 4. It’s evident that the EP/EHBFRs systems exhibit greater improvement in toughness, strength, and *T_g_*. This further manifests the effectiveness of developing high-performance epoxy resins with satisfactory fire safety, toughness, and strength, as well as *T_g_* by incorporating EHBFRs.

## 4. Conclusions

Three kinds of epoxy-terminated hyperbranched flame retardants (EHBFR-HB, EHBFR-HCM, and EHBFR-HBM) containing a rigid central core and branches with varied rigidity were successfully synthesized. All the EHBFRs showed high flame retardancy in cured epoxy systems, and while the P contents in EP/EHBFR-HB, EP/EHBFR-HCM, and EP/EHBFR-HBM were 0.80, 0.73, and 0.75 wt.%, respectively, the EP/EHBFR systems reached the UL-94 V-0 grade. By changing the rigidity of the branched chain in EHBFRs, the *T_g_* of modified epoxy could be adjusted. Therefore, under the combined action of structural rigidity, crosslink density, and free volume, EHBFRs with appropriate branched chain rigidity may give the EP/EHBFRs systems higher *T_g_*. The introduction of EHBFRs can also improve the flexural properties and toughness of cured products. Under the synergistic influence of structure rigidity, crosslink density and free volume, increasing the rigidity of the branched chain in EHBFRs was conducive to improving the flexural strength and modulus, as well as the room temperature impact strength of EP/EHBFRs systems.

## Figures and Tables

**Table 1:** Formulations of different samples.

Samples	Composition (phr)			Content of P (wt.%)
	DGEBA	EHBFR	DDM	
EP	100	0	25.8	0
EP/EHBFR-HB_10_	90	10	23.9	0.26
EP/EHBFR-HB_20_	80	20	22.1	0.53
EP/EHBFR-HB_30_	70	30	20.3	0.80
EP/EHBFR-HCM_10_	90	10	23.8	0.24
EP/EHBFR-HCM_20_	80	20	21.9	0.48
EP/EHBFR-HCM_30_	70	30	20.0	0.73
EP/EHBFR-HBM_10_	90	10	23.9	0.24
EP/EHBFR-HBM_20_	80	20	22.0	0.49
EP/EHBFR-HBM_30_	70	30	20.1	0.75

**Table 2:** Physical parameters of EHBFRs.

Samples	Mn (g/mol)	PDI	DB	EV (mol/100 g)
EHBFR-HB	3872	1.8	0.68	0.15
EHBFR-HCM	4393	1.9	0.72	0.13
EHBFR-HBM	4261	2.0	0.69	0.14

**Table 3:** T_d5_, R_800_, *T_g_*, and physical parameters of epoxy thermosets.

Samples	T_d5_ (°C)	R_800_ (%)	* T_g_* Based on DSC (°C)	* T_g_* Based on DMA (°C)	* Veʹ * (×10^3^ mol·m^−3^)	* α_v,g_* (×10^−6^ K^−1^)	* α_v,r_* (×10^−6^)	Δ*α_v_*_,*r*_ (×10^−6^)
EP	389	14.99	168	172	4.03	63.9	166.0	102.1
EP/EHBFR-HB_10_	380	17.50	160	169	5.04	61.5	171.8	110.3
EP/EHBFR-HB_20_	373	17.85	155	160	5.45	60.8	177.2	116.4
EP/EHBFR-HB_30_	362	19.54	147	151	5.48	60.3	177.4	117.1
EP/EHBFR-HCM_10_	382	16.31	174	183	5.04	61.5	172.4	110.9
EP/EHBFR-HCM_20_	375	18.79	162	173	5.28	60.3	177.8	117.5
EP/EHBFR-HCM_30_	367	19.35	152	161	4.95	59.7	178.7	119.0
EP/EHBFR-HBM_10_	385	16.79	169	179	4.38	60.9	175.7	114.8
EP/EHBFR-HBM_20_	383	22.04	156	167	5.03	59.3	179.0	119.7
EP/EHBFR-HBM_30_	377	23.95	151	160	4.12	58.8	179.7	120.9

**Table 4:** Comparison of increments in toughness (impact strength), strength (the superscript a is flexural strength, the superscript b is tensile strength), and *T_g_* of epoxy thermosets.

Increment in Strength/%	Increment in *T_g_*/%	Increment in Toughness/%	Reference
12.4	−4.3	25	[39] ^a^
3.5	0.3	32.3	[40] ^b^
−2.9	6.7	51	[25] ^a^
22.7	3.1	85.5	[41] ^b^
1.8	−1.8	26.9	[42] ^b^
3.1	2.5	38.2	[43] ^a^ (EP-1)
17.4	0.6	45.4	[43] ^a^ (EP-2)
5.8	1.1	37.7	[44] ^a^ (EP-2)
11.3	0.7	45.9	[44] ^a^ (EP-3)
12.8	−0.6	50.4	[44] ^a^ (EP-4)
15.6	−6.5	63.9	[44] ^a^ (EP-5)
21.4	−13.2	45.5	[45] ^a^ (EP2)
20.4	−9.6	59.1	[45] ^a^ (EP3)
−11.3	−2.9	−13.3	[46] ^b^ (EP-4.0)
−16.7	−3.5	−11.6	[46] ^b^ (EP-7.5)
36	1.4	−29.7	[47] ^a^
37.5	3.1	55.4	[48] ^a^
31.4	−12.2	139.5	EP/EHBFR-HB_30_
34.3	−6.3	88.4	EP/EHBFR-HCM_30_
28.6	−7.0	36.9	EP/EHBFR-HBM_30_

^a^ is flexural strength, ^b^ is tensile strength.

## Data Availability

Not applicable.

## Supplementary Materials

The following supporting information can be downloaded at: https://www.mdpi.com/article/10.3390/polym15020449/s1, Figure S1: ^1^H, ^13^C and 31P NMR spectra, FT-IR spectra and HR-MS spectrum of PDG; Figure S2: DSC curves of EP/ EHBFR-HB systems (a–d) at different heating rates, as well as plots generated following the Kissinger’s (e) and Ozawa’s (f) methods for determination of the activation energy of epoxy thermosets; Figure S3: DSC curves of EP/ EHBFR-HCM systems (a–d) at different heating rates, as well as plots generated following the Kissinger’s (e) and Ozawa’s (f) methods for determination of the activation energy of epoxy thermosets; Figure S4: DSC curves of EP/ EHBFR-HBM systems (a–d) at different heating rates, as well as plots generated following the Kissinger’s (e) and Ozawa’s (f) methods for determination of the activation energy of epoxy thermosets; Figure S5: Raman spectra of EHBFR-HB, EHBFR-HCM, EHBFR-HBM, and pure DGEBA; Figure S6: Non-isothermal TGA and DTG curves for cured EP/ EHBFR-HB (a,b), cured EP/ EHBFR-HCM (c,d), and cured EP/ EHBFR-HBM (e,f); Figure S7: Variation of storage modulus and tanδ with temperature for cured EP/EHBFR-HB (a,b), cured EP/EHBFR-HCM (c,d), and cured EP/ EHBFR-HBM (e,f); Figure S8: Flexural properties (a) and impact strength (b)for epoxy thermosets; Figure S9: HRR (a) and THR (b) curves of epoxy thermosets; Table S1: UL-94 vertical burning and LOI results of epoxy thermosets.
