# Supplementary material for: Synthesis of Hyperbranched Flame Retardants with Varied Branched Chains’ Rigidity and Performance of Modified Epoxy Resins
Source: Polymers (Basel). 2023 Jan 14;15(2):449. doi: 10.3390/polym15020449 (PMC9863857; doi:10.3390/polym15020449)
Supplement: Supplementary file 1 [file polymers-15-00449-s001.zip › polymers-2143057-supplementary.pdf]

Supporting information

The supporting information included **9 Figures and 1 Table**

Number of Page S2–S9: Figure S1–S9.

Number of Page S9: Table S1.

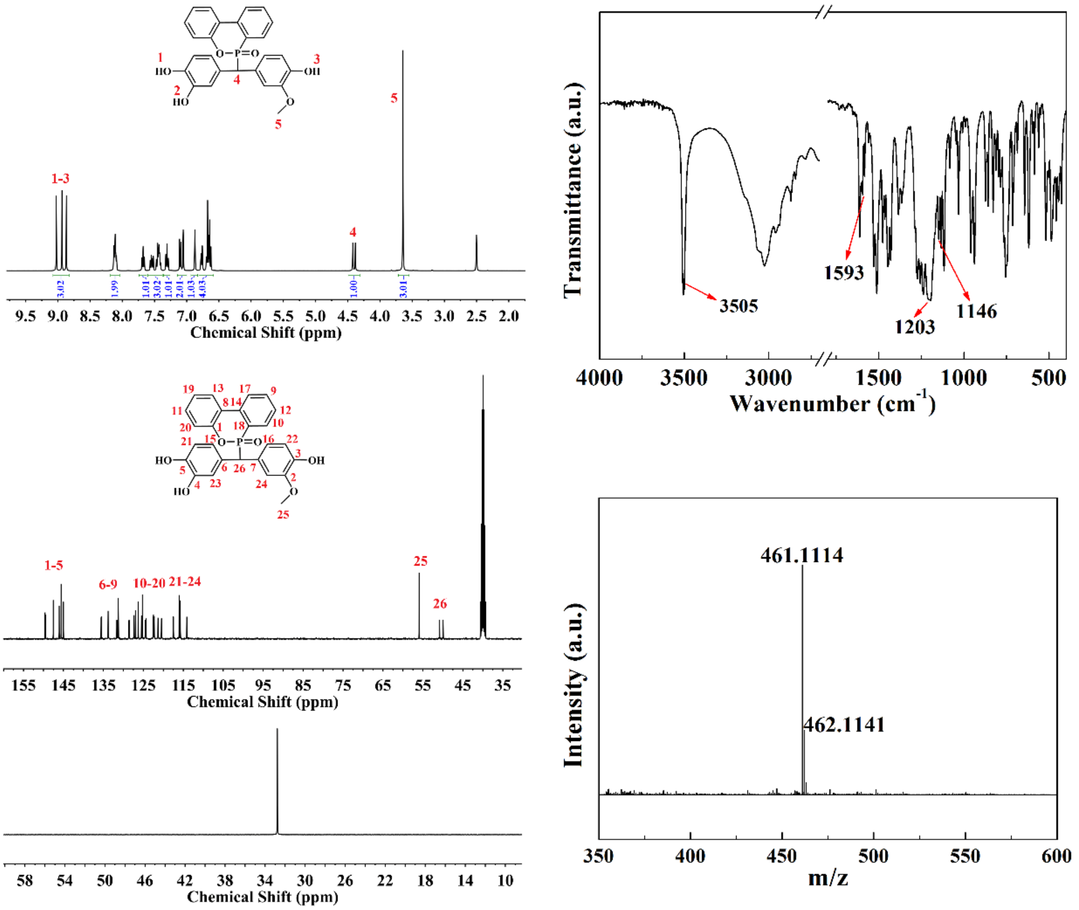

Figure S1. ^{1}H,13C and 31P NMR spectra, FT-IR spectra and HR-MS spectrum of PDG

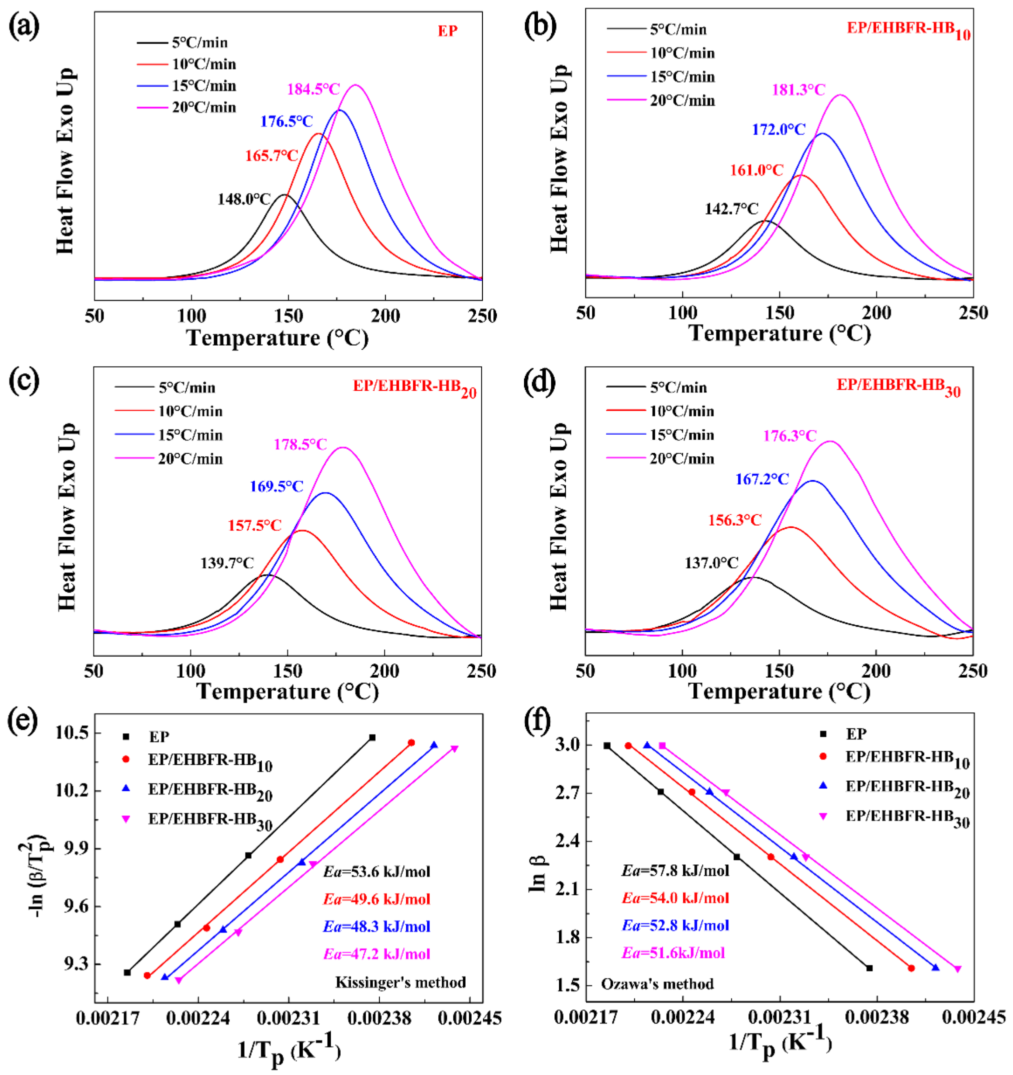

Figure S2. DSC curves of EP/ EHBFR-HB systems (**a**–**d**) at different heating rates, as well as plots generated following the Kissinger's (**e**) and Ozawa's (**f**) methods for determination of the activation energy of epoxy thermosets

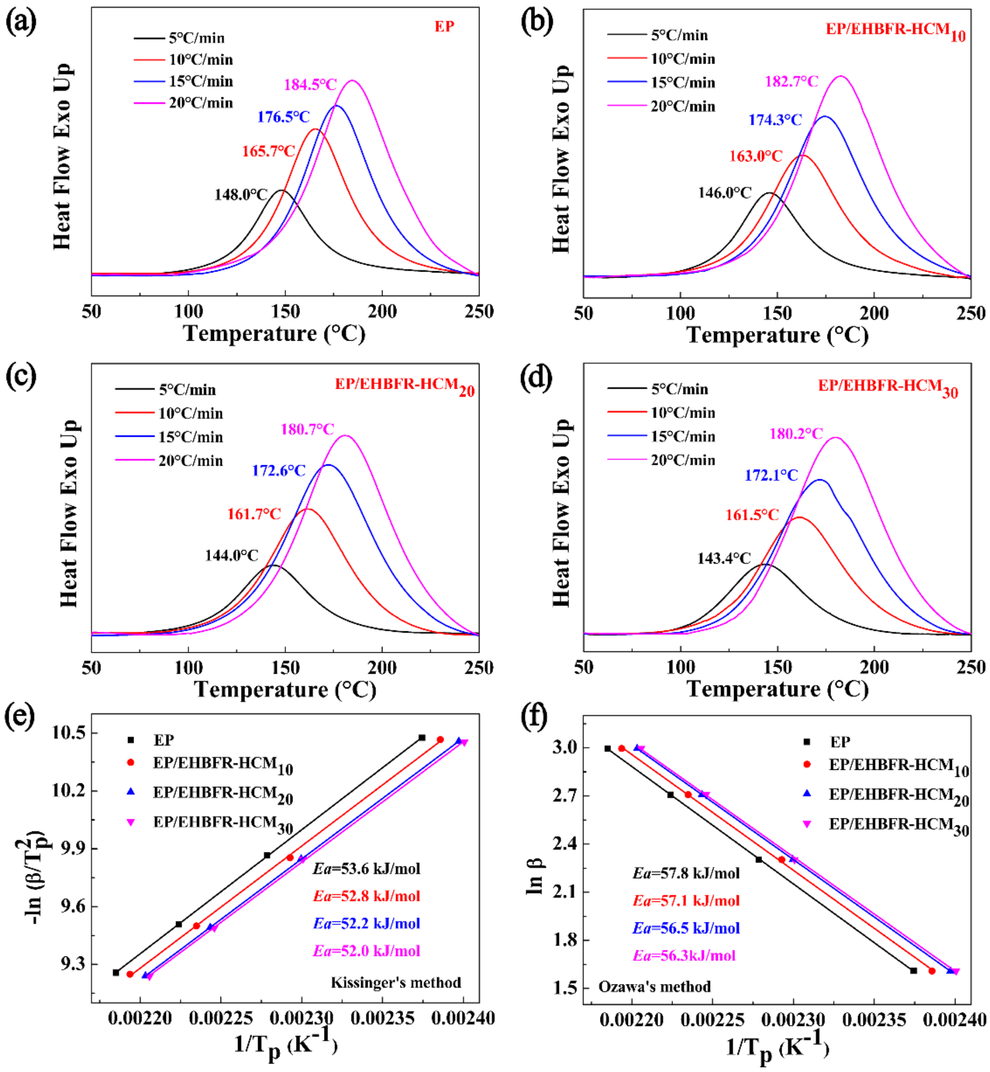

Figure S3. DSC curves of EP/ EHBFR-HCM systems (**a**–**d**) at different heating rates, as well as plots generated following the Kissinger's (**e**) and Ozawa's (**f**) methods for determination of the activation energy of epoxy thermosets

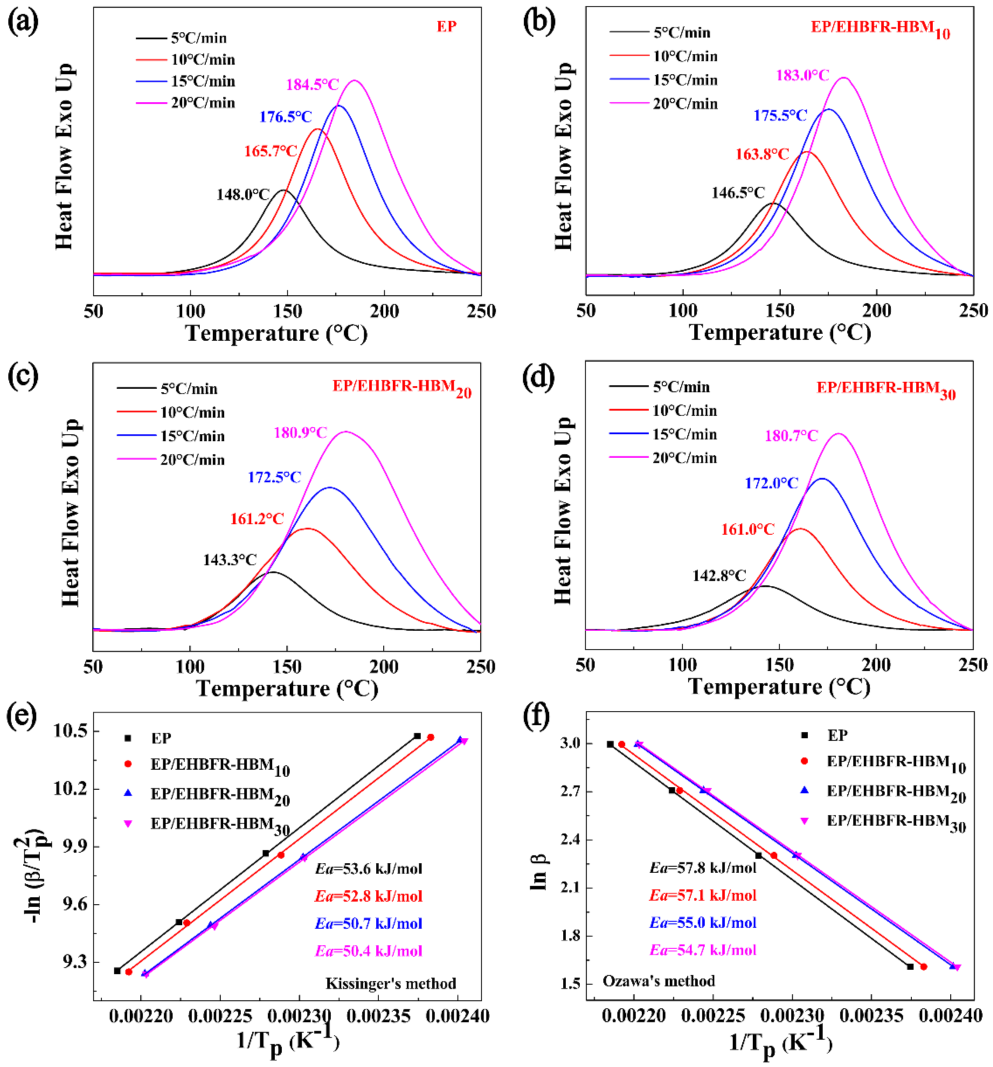

Figure S4. DSC curves of EP/ EHBFR-HBM systems (**a**–**d**) at different heating rates, as well as plots generated following the Kissinger’s (**e**) and Ozawa’s (**f**) methods for determination of the activation energy of epoxy thermosets.

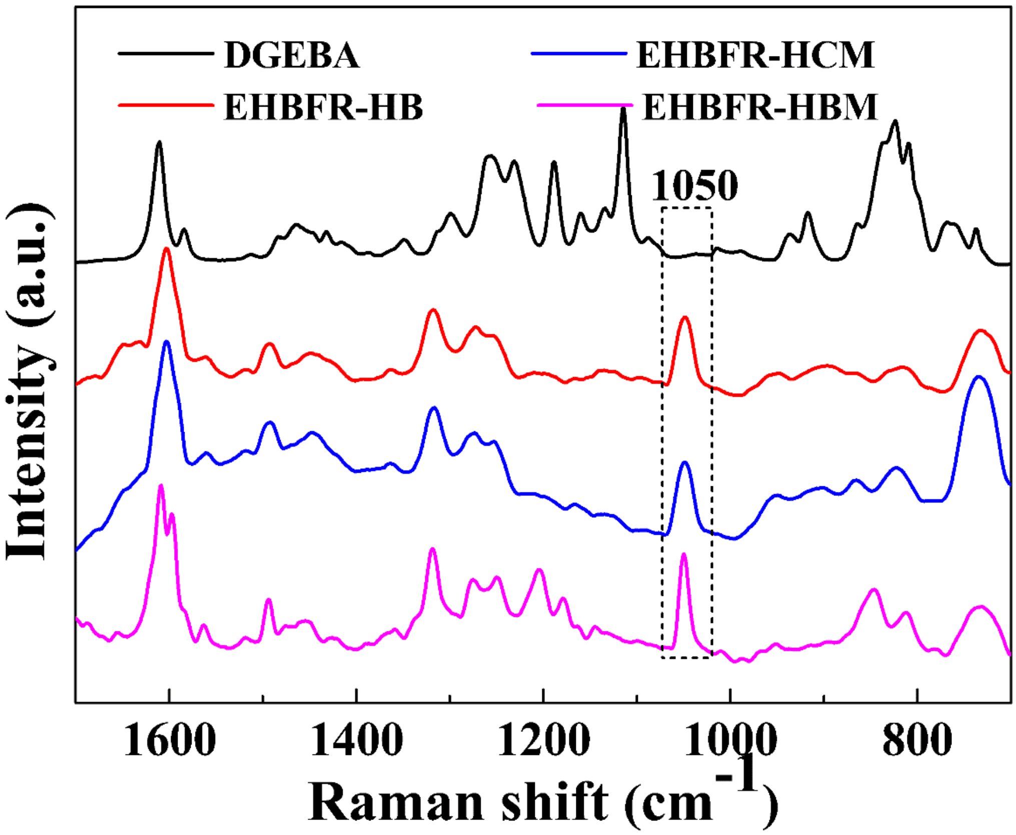

Figure S5. Raman spectra of EHBFR-HB, EHBFR-HCM, EHBFR-HBM, and pure DGEBA

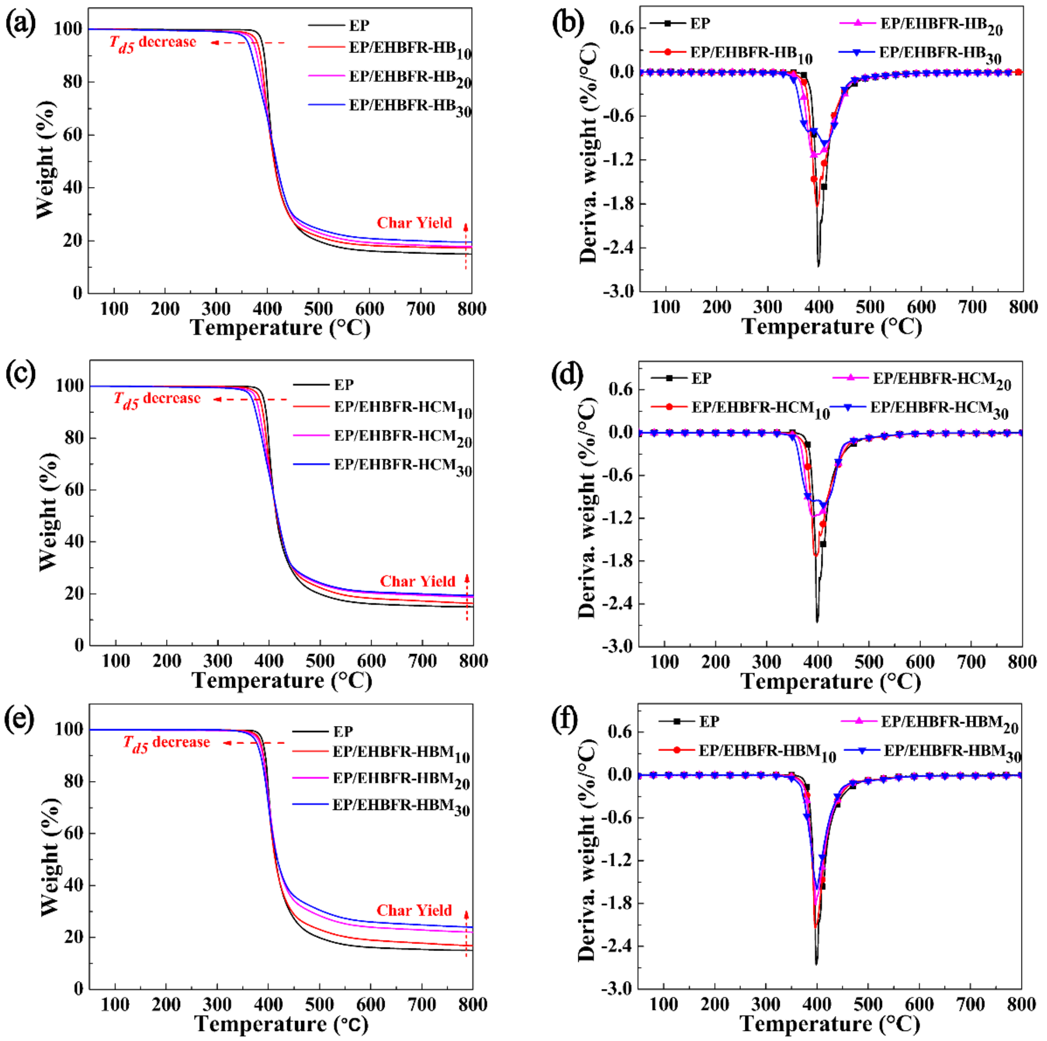

Figure S6. Non-isothermal TGA and DTG curves for cured EP/ EHBFR-HB (**a**,**b**), cured EP/ EHBFR-HCM (**c**,**d**), and cured EP/ EHBFR-HBM (**e**,**f**)

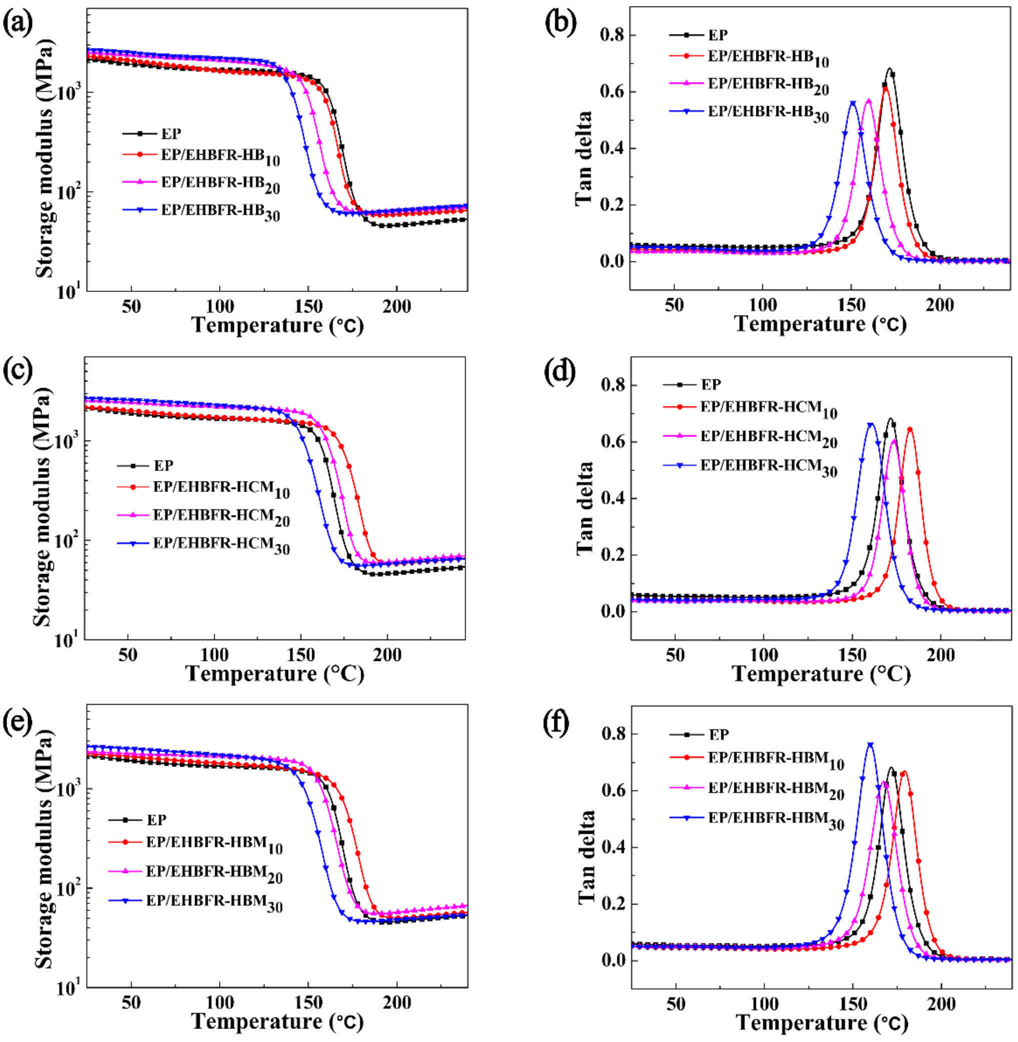

Figure S7. Variation of storage modulus and tanδ with temperature for cured EP/EHBFR-HB (**a**,**b**), cured EP/EHBFR-HCM (**c**,**d**), and cured EP/ EHBFR-HBM (**e**,**f**)

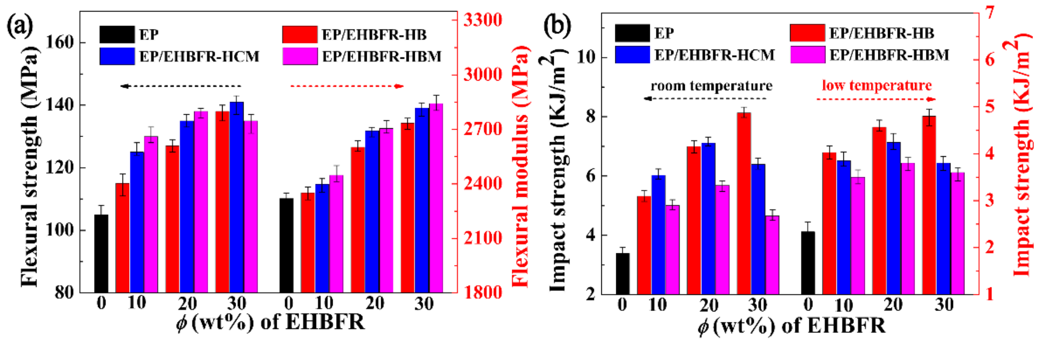

Figure S8. Flexural properties (**a**) and impact strength (**b**)for epoxy thermosets

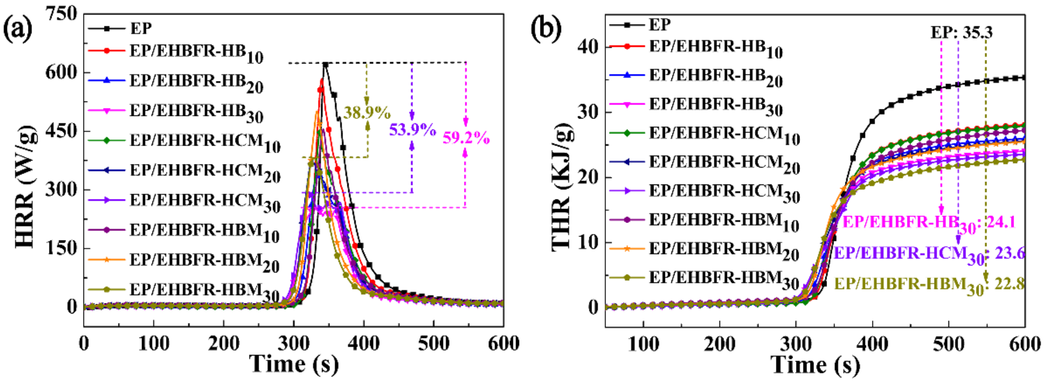

Figure S9. HRR (**a**) and THR (**b**) curves of epoxy thermosets

Table S1. UL-94 vertical burning and LOI results of epoxy thermosets.

Samples	LOI value (vol%)	Vertical burning test			
		After-flame time		Dripping	UL-94 rating
		t_1 (s)	t_2 (s)		
EP	23.6	74.6	–	Yes	No rating
EP/HB$_{10}$	27.1	38.9	–	No	No rating
EP/HB$_{20}$	28.8	7.0	10.8	No	V–1
EP/HB$_{30}$	30.9	3.7	5.3	No	V–0
EP/HCM$_{10}$	27.2	35.4	–	No	No rating
EP/HCM$_{20}$	29.1	9.6	7.8	No	V–1
EP/HCM$_{30}$	30.5	3.4	4.5	No	V–0
EP/HBM$_{10}$	27.3	33.2	–	No	No rating
EP/HBM$_{20}$	29.3	9.6	7.8	No	V–1
EP/HBM$_{30}$	30.7	4.7	2.5	No	V–0
